# Genetic analysis of ATP13A2, PLA2G6 and FBXO7 in a cohort of Chinese patients with early-onset Parkinson’s disease

**DOI:** 10.1038/s41598-018-32217-4

**Published:** 2018-09-19

**Authors:** Ting Shen, Jiali Pu, Hsin-Yi Lai, Lingjia Xu, Xiaoli Si, Yaping Yan, Yasi Jiang, Baorong Zhang

**Affiliations:** 1 Department of Neurology, Second Affiliated Hospital, School of Medicine, Zhejiang University, Hangzhou, 310009 China; 2 Interdisciplinary Institute of Neuroscience and Technology, Qiushi Academy for Advanced Studies, Zhejiang University, Hangzhou, 310029 China

## Abstract

Several genes have already been certified as causative genes in patients with autosomal recessive early-onset Parkinson’s syndrome with pyramidal tract signs, including ATP13A2, PLA2G6 and FBXO7. Variants in these three genes may also play roles in early-onset Parkinson’s disease (EOPD). In order to investigate the contribution of genetic variants in these three genes to Chinese sporadic EOPD patients, we screened 101 Chinese sporadic EOPD patients and 83 age- and sex-matched healthy controls using direct sequencing. Interpretation of those detected variants was performed based on the guidelines developed by the American College of Medical Genetics and Genomics (ACMG). Two missense variants, p.G360E and p.T733M, with “uncertain significance” classification were identified in the ATP13A2 gene and five synonymous variants were significantly over-represented in EOPD patients. Two missense variants, p.R53C and p.T319M, were absent in both our control group and online databases, classified as “likely pathogenic” in the PLA2G6 gene. Only benign variants were identified in the FBXO7 gene. These results indicate that rare variants of PLA2G6 may contribute to PD susceptibility in Chinese population, the ATP13A2 might be associated with higher risk for sporadic EOPD, while the FBXO7 gene doesn’t seem to be a risk factor to develop sporadic PD in Chinese population. Further biochemical and molecular biological studies needs to be conducted to support our main results in our future researches.

## Introduction

Parkinson’s disease (PD) is the second most common neurodegenerative disorder after Alzheimer’s disease and is expected to affect human health seriously, increase the burden of family and society^1^, characterized by complex clinical symptoms including motor symptoms like resting tremor, bradykinesia, rigidity, gait abnormalities and non-motor symptoms like hyposmia, sleep disorders, affective disorders. Onset age of PD less than 50 years was defined as early-onset PD (EOPD) in most literatures, with a prevalence of approximately 5–10% of all the PD patents^2^. The etiology of PD is thought to involve either a genetic predisposition or exposure to an environmental factor, or a combination of both. Compared with late-onset PD (LOPD) population, EOPD appears to form a heterogeneous patient group with a higher proportion of cases due to genetic causes. The identification of different PD-related pathogenic genes and reports of different pathological heterogeneity both have provided evidence that EOPD and LOPD are not the same^3–5^.

Several genes have already been certified as causative genes in patients with autosomal recessive early-onset parkinsonism with pyramidal tract signs, including ATPase 13A2 (ATP13A2), phospholipase A2 group VI (PLA2G6) and F-box protein 7 (FBXO7). The ATP13A2 (PARK9) gene is responsible for Kufor–Rakeb syndrome (KRS), a rare hereditary form of recessive, juvenile-onset, atypical parkinsonism with pyramidal tract signs and cognitive dysfunction^6^. The ATP13A2 gene encodes a lysosomal P-type ATPase, its dysfunction may lead to lysosomal impairment, α-synuclein accumulation and mitochondrial dysfunction those closely associated with PD^7^. Many studies had reported ATP13A2 variants that might leads to higher PD risk^8–10^. The PLA2G6 (PARK14) gene encodes a group VIA calcium-independent phospholipase A2 beta enzyme, which has been proved as a pathogenic factor for neurodegenerative disorders with increased basal ganglia iron accumulation, such as infantile neuroaxonal dystrophy (INAD) and neurodegeneration with brain iron accumulation (NBIA)^11^. Previous studies suggested that this gene was also associated with juvenile-onset dystonia parkinsonism and classic idiopathic PD^12–15^. The FBOX7 (PARK15) gene encodes an E3 ligase, F-box only protein 7, which is involved in ubiquitin proteasome system^16^ and mitochondrial maintenance^17^. Pathogenic variants in FBXO7 gene are likely to cause the EOPD in a similar way to the Parkin and PINK1 genes^17^.

The involvements of these three genes in idiopathic sporadic PD pathogenesis are still unclear. In the present study, we investigated the contribution of genetic variants in ATP13A2, PLA2G6 and FBXO7 to sporadic EOPD patients and complement the current research in China.

## Results

### Genetic results

**Figure 1:**
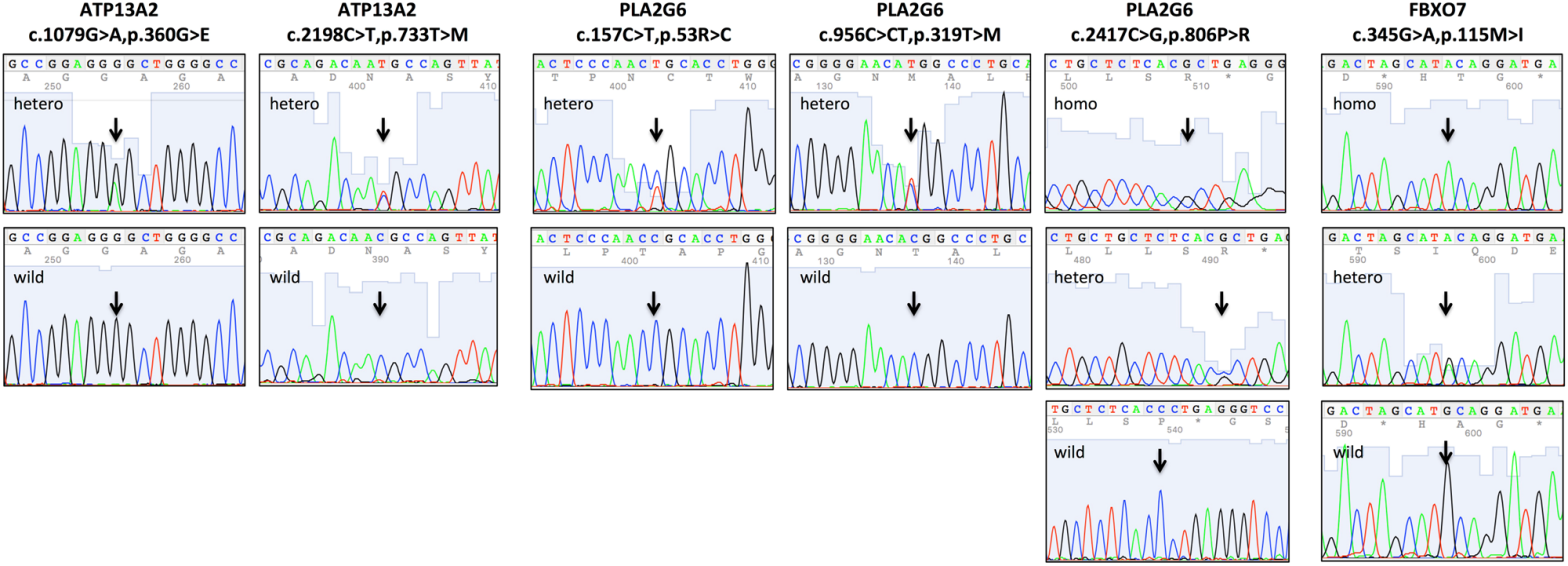
Chromatogram illustrates missense variants of these three genes. Hetero = heterozygous, homo = homozygous, wild = wild type.

**Figure 2:**
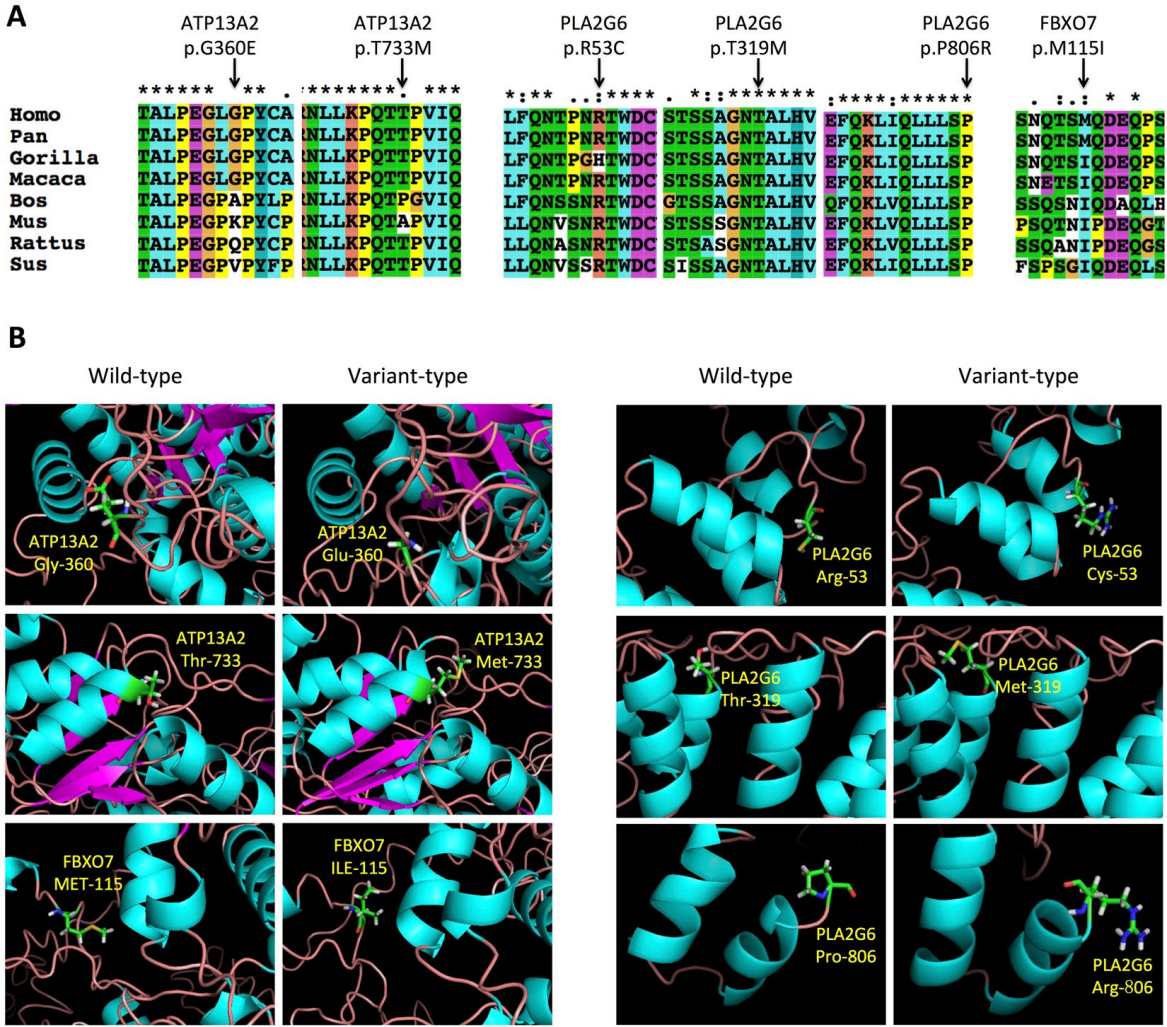
(**A**) Conservation of amino acid residues across different species in three genes. Protein homologs were aligned using ClustalX. Arrows mark the amino acid substitutions identified in this study. Asterisk (*) indicates positions that have a single, fully conserved residue. Colon (:) indicates conservation between groups of strongly similar properties. Period (.) indicates conservation between groups of weakly similar properties. (**B**) The 3-D structures of wild type and variant-type proteins. Protein models were shown in secondary structures. Variant sites were shown in amino acid structures.

We have investigated the exons and exon-intron boundaries of ATP13A2, PLA2G6 and FBXO7 in this study. We found thirteen sequence changes in ATP13A2 gene: (1) two heterozygous missense variants, c.1079G > A variant in exon 12 and c.2198C > T variant in exon 20 (Fig. 1), which caused amino acid change, (2) six synonymous variants and (3) five intron variants of intron/exon boundaries (Table 1). In the PLA2G6 gene, we found fourteen variants: (1) three missense variants all in heterozygous state (Fig. 1), (2) three synonymous variants, including p.L496L in exon 10 that hasn’t been reported previously, but doesn’t lead to amino acid change, (3) eight intron variants of intron/exon boundaries (Table 1). In the FBXO7 gene, we found seven variants: (1) one missense variant in exon 2, c.345G > A (Fig. 1), (2) one synonymous variant in exon 6, p.L317L, (3) five intron variants of intron/exon boundaries, including c.1182 + 133A > G that also hasn’t been detected (Table 1).

**Table 1:** Variants of ATP13A2, PLA2G6 and FBXO7 genes detected in this study.

Variants	Nucleotide change	Protein change	Wild allele (%)	Variant allele (%)	p	Wild genotype (%)	Heterozygote genotype (%)	Homozygote genotype (%)	P for HWE test (PD/Control)	p
			PD/Control	PD/Control		PD/Control	PD/Control	PD/Control		
**ATP13A2**										
rs56047197	c.−227C > T	—	52.4/54.2	47.6/45.8	0.719	31.1/32.5	42.5/43.4	26.4/24.1	0.308/0.516	0.934
rs189404393	c.105 + 24G > A	—	98.2/98.1	1.9/1.9	0.724	96.3/96.2	3.7/3.8	0/0	0.981/0.985	0.721
rs117838709	c.106 − 5C > T	—	97.2/98.0	2.8/2.0	0.882	94.4/96.1	5.6/3.9	0/0	0.957/0.985	0.880
rs566918264	c.1079G > A	p.G360E	99.1/99.4	1.0/0.6	0.813	98.1/98.8	1.9/1.3	0/0	0.995/0.998	0.812
rs117758987	c.1195 + 9C > T	—	96.7/95.0	3.3/5.0	0.421	93.3/90.0	6.7/10.0	0/0	0.939/0.895	0.411
rs9435736	c.1195 + 66A > G	—	20.0/18.1	80.0/81.9	0.650	11.4/2.5	17.1/31.3	71.4/66.3	0.000012*/0.894	0.012*
rs2076603	c.1815C > T	p.P605P	65.6/83.1	34.4/17.0	0.0007*	47.2/72.9	36.8/20.3	16.0/6.8	0.162/0.103	0.0057*
rs201883464	c.2198C > T	p.T733M	99.5/100.0	0.5/0	1.000	99.1/100.0	0.1/0	0/0	0.999/−	1.000
rs9435662	c.2637C > T	p.G879G	65.1/76.3	34.9/23.7	0.021*	46.2/61.5	37.7/29.5	16.0/9.0	0.218/0.263	0.100
rs3738815	c.2790G > A	p.S930S	49.5/41.0	50.5/59.0	0.106	31.1/19.2	36.8/43.6	32.1/37.2	0.025/0.681	0.192
rs761421	c.2970G > A	p.V990V	66.4/79.2	33.6/20.8	0.0085*	47.7/65.3	37.4/27.8	15.0/6.9	0.242/0.408	0.050
rs9435659	c.3192C > T	p.A1064A	65.4/79.2	34.6/20.8	0.005*	46.7/65.3	37.4/27.8	15.9/6.9	0.199/0.408	0.035*
rs3170740	c.3516G > A	p.P1172P	65.9/79.2	34.1/20.8	0.0065*	46.7/65.3	38.3/27.8	15.0/6.9	0.312/0.408	0.039*
**PLA2G6**										
rs2267369	c.87G > A	p.V29V	89.4/87.5	10.6/12.5	0.577	80.6/80.0	17.6/15.0	1.9/5.0	0.735/0.019	0.446
rs370151752	c.157C > T	p.R53C	99.5/100.0	0.5/0	1.000	99.1/100.0	1.0/0	0/0	0.999/−	1.000
rs2267368	c.209 + 16C > T	—	89.4/87.5	10.6/12.5	0.577	80.6/80.0	17.6/15.0	1.9/5.0	0.735/0.019	0.446
rs185396488	c.564C > CT	p.T188T	99.5/100.0	0.5/0	1.000	99.1/100.0	0.9/0	0/0	0.999/−	1.000
rs4375	c.609 + 71A > G	—	63.6/74.4	36.4/25.6	0.026*	47.7/56.3	31.8/36.3	20.6/7.5	0.005*/0.908	0.046*
rs12329956	c.797 + 43C > T	—	86.5/85.1	13.6/14.9	0.707	74.8/74.0	23.4/22.1	1.9/3.9	1.000/0.516	0.700
rs149653983	c.956C > T	p.T319M	99.5/100.0	0.5/0	1.000	99.1/100.0	0.9/0	0/0	0.999/−	1.000
rs2235347	c.1349 − 431T > C	—	85.7/86.7	14.4/13.3	0.770	75.0/76.0	21.3/21.5	3.7/2.5	0.381/0.840	0.904
rs2072867	c.1428 − 70T > C	—	81.3/78.8	18.8/21.2	0.564	69.2/63.0	24.0/31.5	6.7/5.5	0.099/0.884	0.538
none	**c.1488C > G**	p.L496L	99.5/100.0	0.5/0	1.000	99.0/100.0	1.0/0	0/0	0.999/−	1.000
rs4820315	c.1591 + 371G > A	—	85.6/83.6	14.4/16.4	0.604	74.0/72.6	23.1/21.9	2.9/5.5	0.802/0.225	0.681
rs11570751	c.1743 − 26C > T	—	82.4/80.4	17.6/19.6	0.618	70.4/63.3	24.1/34.2	5.6/2.5	0.211/0.759	0.228
rs2076114	c.2202 + 55G > A	—	83.8/79.0	16.2/21.0	0.233	70.4/61.7	26.9/34.6	2.8/3.7	0.993/0.930	0.459
rs140758033	c.2417C > G	p.P806R	99.1/98.0	0.9/2.0	0.680	98.2/96.0	1.9/4.0	0/0	0.995/0.985	0.678
**FBXO7**										
rs369894369	c.122 + 60A > G	—	97.7/98.7	2.3/1.3	0.736	95.4/97.4	4.6/2.6	0/0	0.970/0.993	0.734
rs8136485	c.122 + 116C > T	—	86.6/86.5	13.4/13.5	0.992	76.9/78.2	19.4/16.7	3.7/5.1	0.236/0.042	0.812
rs11107	c.345G > A	p.M115I	33.2/31.9	66.8/68.1	0.801	9.3/10.1	47.7/43.5	43.0/46.4	0.740/1.000	0.863
rs738982	c.872 − 75T > C	—	34.1/33.1	65.9/66.9	0.842	11.2/8.8	45.8/48.8	43.0/42.5	0.981/0.669	0.835
rs9726	c.949C > T	p.L317L	34.1/33.1	65.9/66.9	0.842	11.2/8.8	45.8/48.8	43.0/42.5	0.981/0.669	0.835
none	**c.1182 + 133A > G**	—	98.6/100.0	1.4/0	0.385	97.1/100.0	2.9/0	0/0	0.989/−	0.383
rs5749452	c.1182 + 173T > C	—	34.1/50.0	65.9/50.0	0.0026*	11.2/27.4	45.8/45.2	43.0/27.4	0.981/0.715	0.0097*

Accession number (rs) is given for each known polymorphism. *Means p < 0.05 considered as statistically significant.

### Population databases results

We displayed the distribution of the minor allele frequency (MAF) of these variants in two databases, 1000 Genomes Project (http://www.1000genomes.org/) and ExAC Browser (Exome Aggregation Consortium; http://exac.broadinstitute.org/), which were listed in Table 2. Except c.157C > T, c.564C > T and c.1488C > G in PLA2G6 gene, c.1182 + 133A > G in FBXO7 gene, all other detected variants were found in the 1000 Genomes database. However, in the ExAC database, c.−227C > T and c.1195 + 66A > G in ATP13A2 gene, c.564C > T, c.609 + 71A > G, c.1349-431T > C, c.1428 − 70T > C, c.1488C > G, c.1591 + 371G > A and c.2202 + 55G > A in PLA2G6 gene, c.122 + 60A > G, c.122 + 116C > T, c.872-75T > C, c.1182 + 133A > G and c.1182 + 173T > C in FBXO7 gene were absent. The six variants, c.106-5C > T, c.1079G > A and c.2198C > T in ATP13A2 gene, c.157C > T and c.956C > T in PLA2G6 gene, c.122 + 60A > G in FBXO7 were only found in a heterozygous state with fairly low MAF in the East Asian population.

**Table 2:** Details of the gene variants identified during the present study.

Variants	PhyloP/PhastCons^a^	NNsplice^b^	Protein function prediction				Population MAF (East Asian)^g^		ACMG class^h^
			Polyphen2^c^ (HumDiv)	Mutation Taster^d^	SIFT^e^	Mutation assessor^f^	1000 G	ExAC	
**ATP13A2**									
c.−227C > T	−0.771/0	No	—	P	—	—	0.5496	—	1
c.105 + 24G > A	−0.198/0	DI	—	P	—	—	0.0228^#^	0.0192	1
c.106 − 5C > T	−0.235/0	AMI	—	P	—	—	0.0069^#^	0.0097^#^	1
c.1079G > A	2.984/0.7	DG	benign	DC	T	Low	0.0020^#^	0.0021^#^	3
c.1195 + 9C > T	−1.309/0	No	—	P	—	—	0.0400	0.0095^#^	1
c.1195 + 66A > G	−0.309/0	No	—	P	—	—	0.8194	—	1
c.1815C > T	0.069/0.998	AI, DG	—	P	T	—	0.2589	0.2867	1
c.2198C > T	1.834/0.004	DMI, DI	PsD	P	T	Low	0.0089^#^	0.0042^#^	3
c.2637C > T	−1.168/0.563	No	—	P	T	—	0.2599	0.2900	1
c.2790G > A	−2.84/0.035	AMI, DMI, DI	—	P	T	—	0.5773	0.5525	1
c.2970G > A	−0.51/0.586	DI, DG	—	P	T	—	0.2520	0.2894	1
c.3192C > T	−0.465/0.037	AI, DI	—	P	T	—	0.2599	0.2930	1
c.3516G > A	−1.79/0.006	No	—	P	T	—	0.2460	0.3343	1
**PLA2G6**									
c.87G > A	1.004/0.032	DI	—	P	T	—	0.0992	0.1119	1
c.157C > T	3.092/0.965	DG	PrD	DC	D	Medium	—	—	4
c.209 + 16C > T	−0.703/0	DG	—	P	—	—	0.0992	0.1263	1
c.564C > T	1.15/1	AMI, AG	—	DC	T	—	—	—	3
c.609 + 71A > G	0.09/0.004	AMI, AI, AG	—	P	—	—	0.2887	—	1
c.797 + 43C > T	1.62/0.002	AMI	—	P	—	—	0.1438	0.1545	1
c.956C > T	2.928/0.992	DMI	PrD	DC	T	Medium	—	—	4
c.1349 − 431T > C	−2.412/0	DI	—	P	—	—	0.1984	—	1
c.1428 − 70T > C	−0.287/0	DI	—	P	—	—	0.2272	—	1
c.1488C > G	−0.463/0.82	AMI, DG	—	DC	T	—	—	—	3
c.1591 + 371G > A	−1.18/0	DI	—	P	—	—	0.1438	—	1
c.1743 − 26C > T	−3.604/0	AI, AMI	—	P	—	—	0.2192	0.1982	1
c.2202 + 55G > A	0.494/0	DI, DG	—	P	—	—	0.2192	—	1
c.2417C > G	1.034/0.778	AI, AMI, DI, DMI	PrD	DC	D	Low	0.0149^#^	0.0268	2
**FBXO7**									
c.122 + 60A > G	−0.837/0.013	No	—	P	—	—	0.0079^#^	—	1
c.122 + 116C > T	−0.97/0	DG	—	P	—	—	0.1567	—	1
c.345G > A	−0.862/0	DMI, DI	benign	P	T	Neutral	0.6915	0.6908	1
c.872 − 75T > C	0.492/0.003	AMI, AI	—	P	—	—	0.6925	—	1
c.949C > T	1.317/1	AMI, AI	—	P	T	—	0.6925	0.6896	1
c.1182 + 133A > G	0.005/0.009	DI	—	P	—	—	—	—	1
c.1182 + 173T > C	−0.098/0.005	DI	—	P	—	—	0.4692	—	1

^a^PhyloP values between −14 and +6, PhastCons values between 0 and 1, the closer the value is to maximum, the more probable the nucleotide is conserved; ^b^NNsplice analysis possible changes in splice site, i.e. donor increased (DI), donor gained (DG), Acc marginally increased (AMI), Acc increased (AI), Acc gained (AG); ^c^PolyPhen2 predictions are probably damaging (Pr.D), possibly damaging (PsD) and benign; ^d^MutationTaster predictions are disease causing (DC) and polymorphism (P); ^e^SIFT predictions are deleterious (D) and tolerated (T); ^f^Mutation Assessor predictions are neutral; low impact (Low), medium impact (Medium), high impact (High); ^g^Minor allele frequency (MAF) of the variant allele among the East Asian population, # indicates that only heterozygous was found; ^-^indicates that no variant was found; ^h^ACMG classification: 5 = pathogenic, 4 = likely pathogenic, 3 = uncertain significance, 2 = likely benign and 1 = benign (see Methods).

### Statistical analysis results

Before genetic analysis, it is preferred to check whether our data are free from sample level substructure or genotyping error using Hardy-Weinberg equilibrium (HWE) test. We proved that most genotype frequencies in healthy control group were in agreement with the HWE principle (p > 0.05), the c.2790G > A variant in ATP13A2 gene, c.87G > A and c.209 + 16C > T variants in PLA2G6 gene were also didn’t deviated from HWE principle (p > 0.01). We found the c.1195 + 66A > G variant (p = 0.000012) in ATP13A2 gene and c.609 + 71A > G variant (p = 0.005) in PLA2G6 gene did not fit with HWE principle in EOPD group, indicating these variants might be associated with EOPD (Table 1).

Genotype distribution and allele frequencies of these variants are shown in Table 1. For ATP13A2 gene, the frequencies of T allele in c.1815C > T (p = 0.0007), T allele in c.2637C > T (p = 0.021), A allele in c.2970G > A (p = 0.0085), T allele in c.3192C > T (p = 0.005) and A allele in c.3516G > A (p = 0.0065) were observed significantly higher in EOPD group compared to healthy controls. The frequencies of genotypes of c.1195 + 66A > G (p = 0.012), c.1815C > T (p = 0.0057), c.3192C > T (p = 0.035) and c.3516G > A (p = 0.039) were significantly different between EOPD patients and healthy controls. For PLA2G6 gene, the frequency of G allele in c.609 + 71A > G (p = 0.026) was significantly higher in EOPD group and its frequencies of genotypes (p = 0.046) were significantly different between the two groups. In the case of FBXO7 gene, the frequency of C allele in c.1182 + 173T > C (p = 0.0026) was significantly higher in EOPD group and its frequencies of genotypes (p = 0.0097) were significantly different between EOPD patients and healthy controls. Distributions of genotypes and alleles of other variants showed no significant differences between two groups.

### *In silico* analysis results

Based on the results of multiple sequence alignment carried out by the program ClustalX, p.T319M and p.P806R in the PLA2G6 gene were highly conserved across known mammalian species; p.R53C in the PLA2G6 gene and p.M115I in FBXO7 gene had strongly similar properties between different species; p.T733M in the ATP13A2 gene had weakly similar properties between different species; p.G360E in the ATP13A2 gene was not conserved across listed mammalian species, might be conserved between primate species (Fig. 2A). The phlyoP and phastCons scores were listed in the Table 2, higher score indicating higher conservation.

Pathogenicity of these variants was predicted using PolyPhen-2, Mutation Taster, SIFT scores and Mutation assessor (Table 2). The p.T733M in ATP13A2 gene, p.R53C, p.T319M and p.P806R in PLA2G6 gene were predicted to be probably damaging or possibly damaging by PolyPhen-2. These four variants and p.L496L synonymous variant were predicted to be disease causing, while other detected variants as polymorphism using Mutation Taster program. SIFT program predicted two missense variants, p.R53C and p.P806R in PLA2G6 gene as damaging, while other detected variants as tolerated. The Mutation Assessor program predicted two missense variants as medium impact, three as low and one as neutral.

### Structural prediction

The modeled 3-D structure for wild type and variant type ATP13A2, PLA2G6 and FBXO7 proteins were shown in Fig. 2B. The amino acid changes in different sites were also visualized. From the structural prediction results of the six missense variants, only p.T733M in the ATP13A2 gene was located at an alpha-helix structure, while others were all located at random coils. All these six missense variants didn’t cause obvious secondary structure transformation and solvent accessibility change.

### Expression QTL (eQTL) analysis

**Figure 3:**
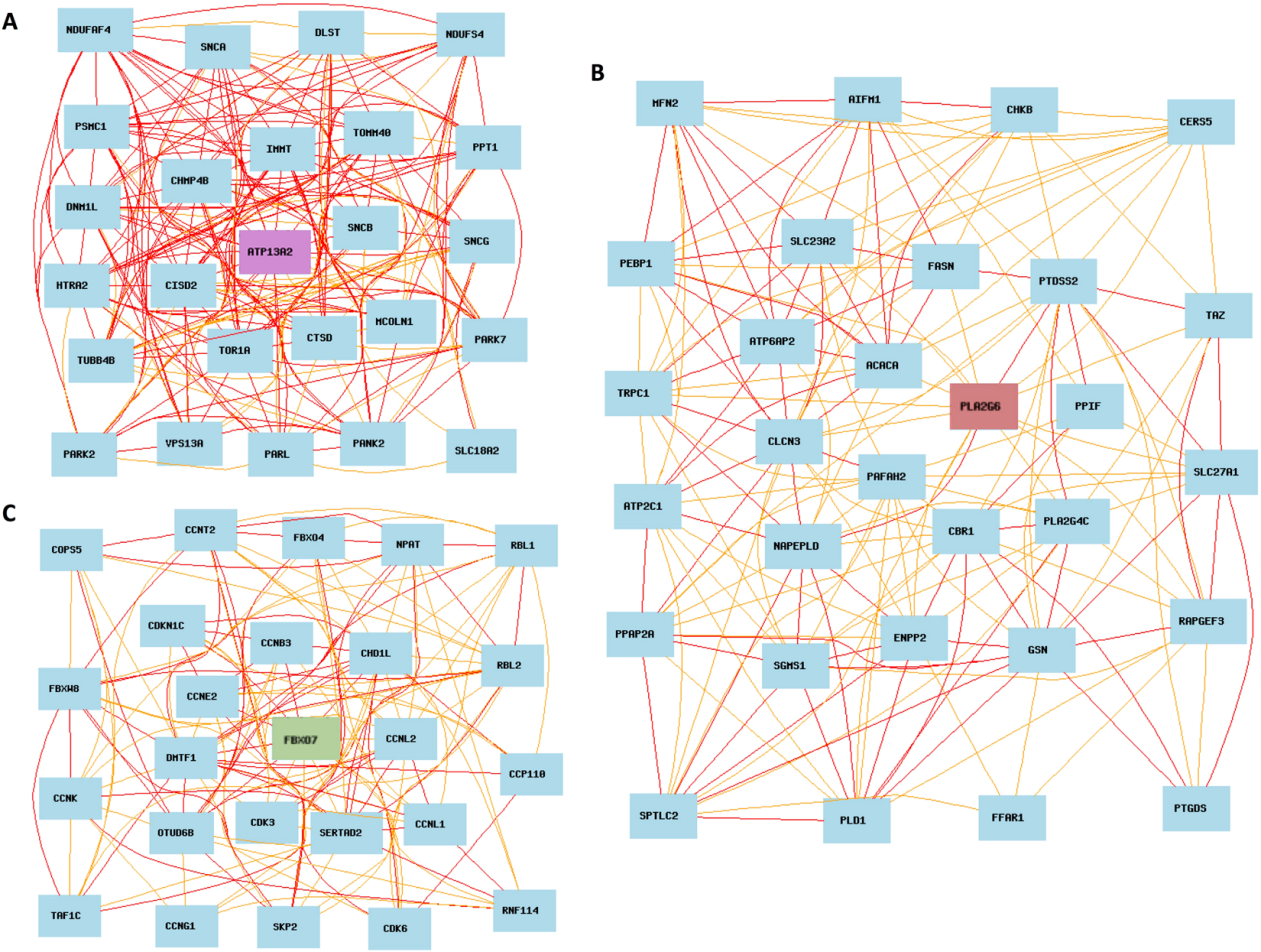
Genetic network co-expression graphs. (**A**) ATP13A2, (**B**) PLA2G6, (**C**): FBXO7. Each node represents a transcript of related gene, red and yellow lines represent Pearson correlation coefficients of 0.7–1.0 and 0.5–0.7 respectively.

To identify biologic relations and the strength of correlation among genes with ATP13A2 or PLA2G6 or FBXO7 is correlated can be evaluated using the co-expressed network. Genes with relatively high correlation coefficient were chosen for gene network construction. In the case of ATP13A2 gene, the graph generated from the wild-type data set showed an ATP13A2-centered graph with 26 genes directly related to ATP13A2 (both the sample p-value and literature p-value > 0.590, Fig. 3A). For the PLA2G6 gene, 29 genes with sample p-value and literature p-value > 0.517 were selected to construct the PLA2G6-centered graph (Fig. 3B). For the FBXO7 gene, an FBXO7-centered graph was generated from 24 genes directly related to FBXO7 (both the sample p-value and literature p-value > 0.500, Fig. 3C).

We obtained the brain expression pattern of ATP13A2 gene from the Braineac database, which showed significant higher expression level (2.6-fold change, p = 5.4 × 10^−56^) in thalamus (THAL) than intralobular white matter (WHMT) (Supplementary Fig. S1A). The effect of several variants on the expression level of ATP13A2 in Braineac showed no significant difference among different genotypes (Supplementary Fig. S2A). For the PLA2G6 gene, significant regional expression difference was observed, with WHMT showing the highest expression and cerebellum (CRBL) showing the lowest expression (Supplementary Fig. S1B). Significant higher expression was found in OCTX of rs4375, rs223534347, rs4820315 and rs12329956 variant genotypes (Supplementary Fig. S2B). In the case of the FBXO7 gene, we also found significant regional expression difference with highest expression in WHMT and lowest expression in CRBL (Supplementary Fig. S1C). Variant-type alleles of rs9729, rs11107 and rs738982 were associated with significant higher expression level of FBXO7 in WHMT and medulla inferior olivary nucleus (MEDU) (Supplementary Fig. S2C).

## Discussion

**Figure 4:**
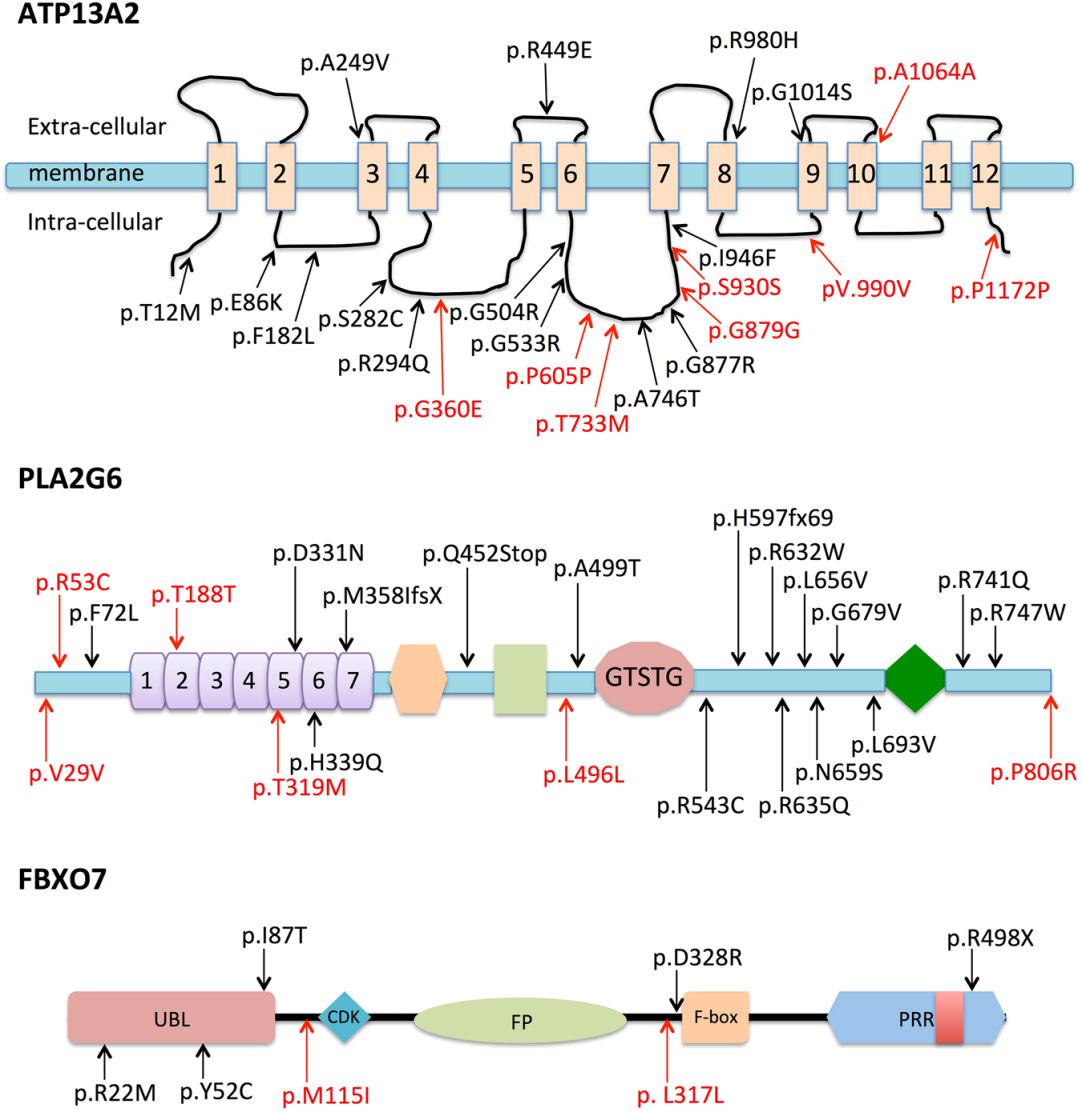
Schematic of gene coding regions and locations of variants detected in these genes. Variants are shown above the schematic. Black arrows indicate previous identified variants in PD and red arrows show variants detected in our study.

Previous studies showed decreased 99mTc-TRODAT-1 uptakes in striatum of patients carried ATP13A2 variants^10^. Santoro *et al*.^18^ performed DaTSCAN SPECT imaging that showed marked defects of the nigrostriatal dopaminergic systems. Molecular and cellular experimental results showed several variants impaired the protein stability of ATP13A2, enhanced its degradation by the proteasome and disrupted the localization of ATP13A2^18^. In our present study, we found two missense variants (p.G360E and p.T733M), six synonymous variants (p.P605P, p.G879G, p.S930S, p.V990V, p.A1064A and p.P1172P) and five intron variants (c.−227C > T, c.105 + 24G > A, c.106 − 5C > T, c.1195 + 9C > T and c.1195 + 66A > G) in ATP13A2 gene (Fig. 4A). Synonymous variants and intron variants that all have relatively high MAF (MAF > 0.01) in the East Asian population (in the 1000 Genomes or ExAC database), predicted to be “polymorphism” by *in silico* predictive algorithms. Nonetheless, the frequencies of variant alleles of synonymous variants were significantly higher in our EOPD group, indicating higher EOPD risk. However, all synonymous, intron variants were found to be “benign”, according to the guidelines developed by the ACMG for the interpretation of sequence variants. The missense variant p.G360E is located at a relatively conserved region with fairly low MAF (0.00198 in the 1000 Genomes database and 0.00209 in the ExAC database). The p.G360E variant was not only observed in EOPD patients, we also found one heterozygous carrier in our control group. It was predicted to be “disease causing” by Mutation Taster, “low impact” by Mutation assessor, whereas it was predicted to be “benign” by PolyPhen-2, “tolerated” by SIFT and. The missense variant p.T733M is located at a weakly conserved region with fairly low MAF (0.00893 in the 1000 Genomes database and 0.00417 in the ExAC database). The p.T733M variant was only observed in EOPD patients. It was predicted to be “possibly damaging or benign” by PolyPhen-2, “low impact” by Mutation assessor, whereas it was predicted to be “polymorphism” by Mutation Taster and “tolerated” by SIFT. These two missense variants detected in our study haven’t been previously reported in EOPD patients. They were both classified as “Uncertain significance” by ACMG criteria and their contributions to PD risk still need to be studied. The ATP13A2 transcript showed different levels in different brain region, thalamus, cortex and substantia nigra being the highest. These three regions are included in the cortex-basal ganglia-thalamus circuit, which is of particular relevance to PD. The ATP13A2-centered graph showed high correlation coefficient with known PD-related genes, such as SNCA, PARK2 and PARK7. These results indicate that the ATP13A2 might be associated with higher risk for EOPD in Chinese population.

Based on previous researches, enzyme activity of variant PLA2G6 was found to be decreased compared to wild-type PLA2G6^15^. Postmortem examination of one patient with PLA2G6 gene variant showed Lewy bodies (LDs) in the substantia nigra and locus ceruleus, excessive iron accumulation was also presented in the substantia nigra pars reticulata, globus pallidus and ventral forebrain^19^. Miki *et al*.^20^ found that PLA2G6 accumulated in LDs in both PLA2G6-related PD and idiopathic PD, demonstrating regulating effect of PLA2G6 in the formation of LDs. Here, we found three missense variants (p.R53C, p.T319M and p.P806R), three synonymous variants (p.V29V, p.T188T and p.L496L) and eight intron variants (c.209 + 16C > T, c.609 + 71A > G, c.797 + 43C > T, c.1349 − 431T > C, c.1428 − 70T > C, c.1591 + 371G > A, c.1743 − 26C > T and c.2202 + 55G > A) in PLA2G6 gene (Fig. 4B). Previous known synonymous variants and intron variants all have relatively high MAF (MAF > 0.01) in online databases, predicted to be “polymorphism” by *in silico* predictive algorithms. The missense variants p.R53C and p.T319M were both located at highly conserved region and absent in both two databases in the East Asian population. These two variants haven’t been previously reported in EOPD patients and only observed in EOPD patients in our study. Both two variants were predicted to be “disease causing” by Mutation Taster, “probably damaging” by PolyPhen-2 and “low or medium impact” by Mutation assessor. They were both classified as “Likely pathogenic” by ACMG criteria after manually adjustment. The missense variant p.P806R has been previously reported in PD patients, which is located at a relatively conserved region with fairly high MAF (0.01488 in the 1000 Genomes database and 0.02678 in the ExAC database). Although it was predicted to be “disease causing” by Mutation Taster, “probably damaging” by PolyPhen-2 and “damaging” by SIFT, this variant was also observed in three control subjects. The heterozygous p.P806 variant had been detected in EOPD patients before^14,21^. Hattori *et al*.^21^ performed a case–control study suggested that the p.P806 wasn’t a disease-associated variant in PD in Japanese population. Taken together, the p.P806R variant was classified as “Likely benign”. The synonymous variants p.T188T and p.L496L were absent in the both two databases and predicted to be “disease causing” by Mutation Taster. We also didn’t find these two variants in our control group. These two variants were found to be “Uncertain significance” based on the ACMG guidelines. The PLA2G6 transcript showed highest expression level in white matter and slightly lower levels in cortex, thalamus, substantia nigra and putamen. The rs4375, rs223534347, rs4820315 and rs12329956 variants of PLA2G6 gene altered the expression level in occipital cortex. The PLA2G6-centered graph showed high correlation coefficient with known PD-related genes, such as SNCA, PARK2 and PARK7. Taken together, our results supported that rare variants of PLA2G6 may contribute to PD susceptibility in Chinese population.

We found one missense variant (p.M115I), one synonymous variant (p.L317L) and five intron variants (c.122 + 60A > G, c.122 + 116C > T, c.872 − 75T > C, c.1182 + 133A > G and c.1182 + 173T > C) in the FBXO7 gene (Fig. 4C). The intron variant c.1182 + 133A > G was first found and absent in the online database and our control group. It was predicted to be “polymorphism” by Mutation Taster. Other variants were all present in both databases with relatively high MAF. All variants in the FBXO7 gene were found to be “benign” based on the ACMG guidelines. Luo *et al*.^22^ found the p.M115I variant in FBXO7 gene, which was considered to be a polymorphism. Their study suggested that FBXO7 pathogenic variants might be rare in Chinese EOPD patients. Lin *et al*.^23^ also considered FBXO7 gene as a low risk factor to PD. The FBXO7 transcript showed highest expression level in white matter and medulla inferior olivary nucleus, with significant higher expression caused by rs9729, rs11107 and rs738982 variants. Taken together, the FBXO7 gene doesn’t seem to be a risk factor to develop PD in Chinese population.

In conclusion, our results are complement and amendment for the contribution of genetic variants in the ATP13A2, PLA2G6 and FBXO7 genes to Chinese sporadic EOPD patients. ATP13A2 and PLA2G6 genes are associated with increased risk of EOPD, while the FBXO7 gene doesn’t seem to be a risk factor to develop PD in Chinese population. However, further genetic analysis needs to be carried out with a larger population of subjects and the pathological characteristics of genetic variants also needs to be investigated by *in vitro* or *in vivo* functional study.

## Materials and Methods

### Subjects

In this study, 101 idiopathic sporadic EOPD patients with mean age of 52.3 ± 1.6 years (age at onset 43.82 ± 7.8, 48 male and 53 female) and 83 age- and sex-matched healthy controls (mean age 52.8 ± 0.9 years, 44 male and 39 female) were included. All subjects were recruited from the outpatient clinic of the Department of Neurology, Second Affiliated Hospital, School of Medicine, Zhejiang University of China and diagnosed with The International Parkinson and Movement Disorder Society (MDS) clinical diagnostic criteria for PD^24^. Patients with evidence of secondary parkinsonism caused by other neurological diseases or history of any other major disease (such as diabetes, hypertension, cardiovascular/renal/hepatic impairment and *et al*.) were excluded. This study has been approved by ethics committee of Second Affiliated Hospital, School of Medicine, Zhejiang University. Informed consent was obtained from patients who provided blood sample. All the experiments were performed in accordance with approved guidelines.

### Mutation analysis

Approximately 4-6 ml blood sample was collected in vacutainer tubes from patients and genomic DNA was extracted from peripheral blood obtained by Phenol chloroform method. Standard extracted DNA samples had a 260/280 ratio of 1.7–1.9 and concentrations >10 ng/μl. The samples were stored at −20 °C. We designed specific primers (Supplementary Table S1) based on the ATP13A2, PLA2G6 and FBXO7 sequences. Exons and intron/exon boundaries of the three genes were amplified by polymerase chain reaction (PCR). All PCR products were directly sequenced by the Sanger method using an ABI 3730 XL genetic analyzer (Applied Biosystems, Foster City, USA). Alignment and analysis of the sequencing results was carried out with DNAStar (DNAStar, In Madison, WI).

### Statistical analysis

Statistical analysis was performed using the GraphPad prism software. Hardy-Weinberg equilibrium test was used to test for the population stratification and other forms of non-random mating. The chi-squared test was used to analyze allele and genotype distribution between the EOPD and healthy control groups and the results were considered as statistically significant if p < 0.05.

### *In silico* prediction analysis

To evaluate the pathogenicity of genetic variants, *in silico* prediction analysis was performed based on the ACMG guideline^25^. Multiple sequence alignment of ATP13A2, PLA2G6, and FBXO7 protein sequences from different species was performed by ClustalX program, and polyoP/phastCons scores were also obtained to test the gene evolutionary conservation. To predict the effect of variants on gene function, we used four *in silico* algorithms: PolyPhen-2 (http://genetics.bwh.harvard.edu/pph2/)^26^, Mutation Taster (http://www.mutationtaster.org)^27^, SIFT (http://sift-dna.org)^28^ and Mutation assessor (http://mutationassessor.org/r3/). Possible changes of the splice sites were predicted using NNSplice program encoded in Mutation Taster website. Different algorithms may have different performance and features due to their different criteria to assess the predicted impact of a variant, including the evolutionary conservation and biochemical consequence, so we used the multiple prediction software to comprehensively predict the site pathogenicity and we chose the “predictive” results based on the majority rule. 3-D protein structures of wild type and variant type ATP13A2, PLA2G6 and FBXO7 proteins were predicted using an *ab initio* modeling server, I-TASSER program (https://zhanglab.ccmb.med.umich.edu/I-TASSER-MR/)^29,30^. The resulting 3-D models were viewed and edited using the molecular visualization system PyMOL (The PyMOL Molecular Graphics System, Version 1.5, Schrödinger, LLC). Each missense variant was further classified as ‘Benign’ (class 1), ‘Likely benign’ (class 2), ‘Uncertain significance’ (class 3), ‘Likely pathogenic’ (class 4) and ‘Pathogenic’ (class 5) using the automated pathogenicity tool, InterVar software (http://wintervar.wglab.org/), which can generate the preliminary interpretation of the ACMG criteria (only non-synonymous variants have automated ACMG interpretation so far)^31^. Other variants were manually classified into five classes according to ACMG criteria.

### Expression QTL (eQTL) analysis

QTL analysis was carried out using the GeneNetwork programe (http://www.genenetwork.org) to determine co-expression networks^31^. The main pathological characteristic of PD is the death of dopaminergic neurons in the substantia nigra, so we searched the GTEXv5 Human Brain Substantia Nigra RefSeq (Sep15) RPKM log2 Database on GeneNetwork website for ATP13A2, PLA2G6 and FBXO7 genes. The genetic correlation between the expression of these three genes and their related genes were graphically evaluated based on the Pearson correlation coefficients. Genetic co-expression network generated from genes with relatively high correlation coefficient. Genetic regulation of both wild-type and variant-type genes were determined using the Brain eQTL Almanac (Braineac) dataset (http://caprica.genetics.kcl.ac.uk/BRAINEAC/), which contains brain tissues from ten regions including thalamus (THAL), frontal cortex (FCTX), substantia nigra (SNIG), temporal cortex (TCTX), medulla inferior olivary nucleus (MEDU), hippocampus (HIPP), occipital cortex (OCTX), putamen (PUTM), intralobular white matter (WHMT) and cerebellum (CRBL).

## Electronic supplementary material

Supplementary information
