# Supplementary material for: Genetic analysis of ATP13A2, PLA2G6 and FBXO7 in a cohort of Chinese patients with early-onset Parkinson’s disease
Source: Sci Rep. 2018 Sep 19;8:14028. doi: 10.1038/s41598-018-32217-4 (PMC6145881; doi:10.1038/s41598-018-32217-4)
Supplement: Supplementary file 1 — Supplementary information [file 41598_2018_32217_MOESM1_ESM.pdf]

Supplementary Materials

Genetic analysis of ATP13A2, PLA2G6 and FBXO7 in a cohort of Chinese patients with early-onset Parkinson's disease

Ting Shen[1,2], Jiali Pu[1], Hsin-Yi Lai[2], Lingjia Xu[1], Xiaoli Si[1],Yaping Yan[1], Yasi Jiang[2], Baorong Zhang[1,*]

Supplementary table S1. Primer sequences for mutation screening

ATP13A2			
Exons	PCR primers	Exons	PCR primers
Exon1	F:5’>AGGAGCAGGCGGGGACTACA<3’<br>R:5’>CCAAGGGGTGACGACAACTGG<3’	Exon13/15	F:5’>CTTCCCTGCCTTGGGGTC<3’<br>R:5’>TGAGAGAATAACGCGGGTGT<3’
Exon2/3	F:5’>GTGCCTCCCCAAATGACCTT<3’<br>R:5’>GCTGAGGTCCAGAGAAGAGC<3’	Exon16	F:5’>TAGACAGAGAGACGGTGGGG<3’<br>R:5’>AGGGAAGACAGGGTGGGATT<3’
Exon4/6	F:5’>TCTTCTGAGACTGAGTCCATACC<3’<br>R:5’>AGGTTTCAGACAGCAAAAGCATC<3’	Exon17/20	F:5’>TCTGTGAGCCAAAGCCCC<3’<br>R:5’>GCCCAAAAAGATGCCCCAAAA<3’
Exon7/8	F:5’>TTCAAGGGGGCTTACTTGCT<3’<br>R:5’>GGTCTGGTTGCCACCGTAAA<3’	Exon21/23	F:5’>ACGGCTCCAGGTTCTCCTAA<3’<br>R:5’>CGAACGGACAAGCTCAGTCT<3’
Exon9/11	F:5’>GGCTCCAGTCCTTTGGCAG<3’<br>R:5’>CAAAGGGTTGGATGGCAGGG<3’	Exon24/25	F:5’>CAGGCACCATGTGGCATTAG<3’<br>R:5’>TCCGTGCCTGGTGTTTTTCT<3’
Exon12	F:5’>CCTGCTTCCAGGAGTGTCTC<3’<br>R:5’>GATGGGAATGCTCAGACCCC<3’	Exon26/29	F:5’>CCCATCAGATTCCTTCGGGG<3’<br>R:5’>AGGGAGTTCCAGTGTCTGGG<3’
PLA2G6			
Exons	PCR primers	Exons	PCR primers
Exon1	F:5’>GGGGGACAGCCTTCTAGGTA<3’<br>R:5’>ACTGATGGGCCAGAAGTGTG<3’	Exon9	F:5’>CTGGGATCAGGACGAGGGAT<3’ <3’<br>R:5’>CCCAGCATTAATGAACGAGCG<3’ <3’
Exon2	F:5’>GCAGCCTGGGGACCTTC<3’<br>R:5’>ACTATGGAGGGGAACCGAGG<3’	Exon10	F:5’>TAGGACAGAAGTTCCTCGGGT<3’<br>R:5’>AAATGAAGTGTGCAGGCGAG<3’
Exon3	F:5’>AGTCCGAGTTTCCGAGTGC<3’<br>R:5’>AGCAAAGAGACTGAGGACGTG<3’	Exon11	F:5’>TTAGGCCTCGGTAAACCCG<3’<br>R:5’>GTCATTTGAAAGGCCAGTGGG<3’
Exon4	F:5’>AGAATCATTCCCACCTGGACCC<3’<br>R:5’>AGATCTATGGTGGATACTGCTTGC<3’	Exon12	F:5’>CCTATCCCGAACAGAGGTTGG<3’<br>R:5’>CTCTGGCTAGTTCGTCCTGG<3’
Exon5	F:5’>TTCCCACTCTGTCATACTGCTTC<3’<br>R:5’>ATCCCAGCTCTTCATGGACTT<3’	Exon13	F:5’>GTCCCTGGAAAGTCCTCAGC<3’<br>R:5’>GGTCCCTAGCATGGTTTGCT<3’
Exon6	F:5’>CCCAGTACCTGTAGGCCTCT<3’<br>R:5’>AAGGGAAGCAGGATGCTCAC<3’	Exon14	F:5’>ATGCTCTTGCCACCAGAGAC<3’<br>R:5’>GGATCCGCAGCTAAAAAGCG<3’
Exon7	F:5’>GCCTGGGTTCTTACACCCTC<3’<br>R:5’>ACGGGAGAGGATGCTGGTAT<3’	Exon15/16	F:5’>TCAGCCTGACTCGAAAGAGC<3’<br>R:5’>GGCTCTAGACTTTCCCAGCC<3’
Exon8	F:5’>TGGCTGGCTCATTAGTCCCT<3’<br>R:5’>CCTAGAGGCTGACAACTCCG<3’		
FBXO7			
Exons	PCR primers	Exons	PCR primers
Exon1	F:5’>GGCGCTCTATTCCAGAGACC<3’<br>R:5’>TCCCTGGTTCCGTTTACTGC<3’	Exon6	F:5’>GCTAGAGAGGTTGGGCACAG<3’<br>R:5’>ACATTTCGCCAGGAGCAGAA<3’
Exon2	F:5’>AGGGTAATGCGTGCCTAT<3’<br>R:5’>GAGAATCGCTTGAACTGG<3’	Exon7	F:5’>ACAGAAGAGACCATTGGCTAGT<3’<br>R:5’>GCCAGCTCGTTTCTACGTCA<3’
Exon3	F:5’>AGGGAGGGAGGAGTCTAGGA<3’<br>R:5’>CTGCCACTCTCAGACTACGG<3’	Exon8	F:5’>CCATGTAGCACAGAGCAGCA<3’<br>R:5’>ATGCAGCCACCTTCATCAGT<3’
Exon4	F:5’>AAGAGGACTGTGTGGAGTGATTA<3’<br>R:5’>GCCAATCACTGAAGTGGCTAT<3’	Exon9	F:5’>CTTGGTTTATTGCATTGGTTTGGG<3’<br>R:5’>ACTATTCCCAAGGCCAACCT<3’
Exon5	F:5’>ATAAGCTGCTGCCTAGTGGAT<3’<br>R:5’>CACAGATCCCCTCCATATTGGTT<3’		

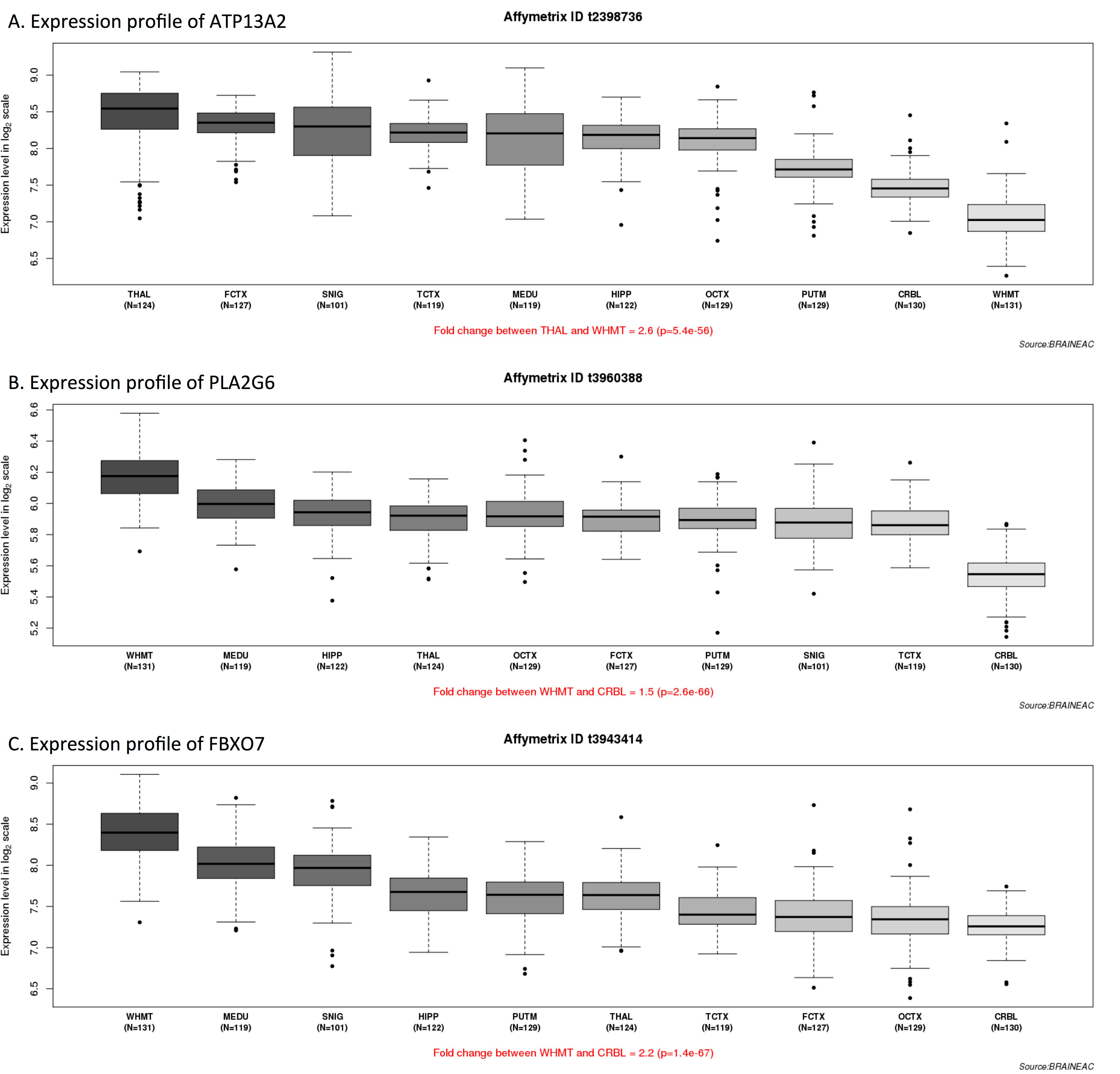
A. Expression profile of ATP13A2
Affymetrix ID t2398736
Expression level in log$_2$ scale
9.0
8.5
8.0
7.5
7.0
6.5
THAL (N=124)
FCTX (N=127)
SNIG (N=101)
TCTX (N=119)
MEDU (N=119)
HIPP (N=122)
OCTX (N=129)
PUTM (N=129)
CRBL (N=130)
WHMT (N=131)
Fold change between THAL and WHMT = 2.6 (p=5.4e-56)
Source:BRAINEAC
B. Expression profile of PLA2G6
Affymetrix ID t3960388
Expression level in log$_2$ scale
6.6
6.4
6.2
6.0
5.8
5.6
5.4
5.2
WHMT (N=131)
MEDU (N=119)
HIPP (N=122)
THAL (N=124)
OCTX (N=129)
FCTX (N=127)
PUTM (N=129)
SNIG (N=101)
TCTX (N=119)
CRBL (N=130)
Fold change between WHMT and CRBL = 1.5 (p=2.6e-66)
Source:BRAINEAC
C. Expression profile of FBXO7
Affymetrix ID t3943414
Expression level in log$_2$ scale
9.0
8.5
8.0
7.5
7.0
6.5
WHMT (N=131)
MEDU (N=119)
SNIG (N=101)
HIPP (N=122)
PUTM (N=129)
THAL (N=124)
TCTX (N=119)
FCTX (N=127)
OCTX (N=129)
CRBL (N=130)
Fold change between WHMT and CRBL = 2.2 (p=1.4e-67)
Source:BRAINEAC

Supplementary Fig.1. Regional distribution of mRNA expression patterns of the three transcripts (ATP13A2, PLA2G6 and FBXO7) in Braineac database. Box plot of mRNA expression levels for these three genes in ten brain regions. Abbreviations: THAL, thalamus; FCTX, frontal cortex; SNIG, substantia nigra; TCTX, temporal cortex; MEDU, medulla inferior olivary nucleus; HIPP, hippocampus; OCTX, occipital cortex; PUTM, putamen; WHMT, intralobular white matter; CRBL, cerebellum.

A. ATP13A2 expression stratified by different variations

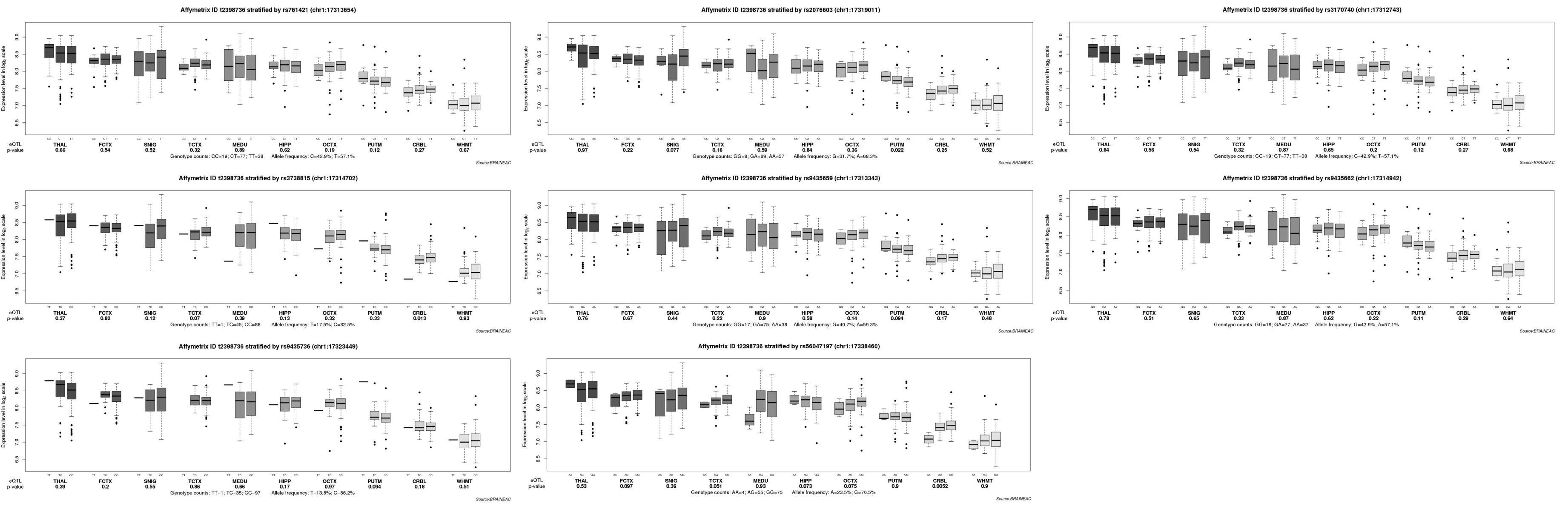

B. PLA2G6 expression stratified by different variations

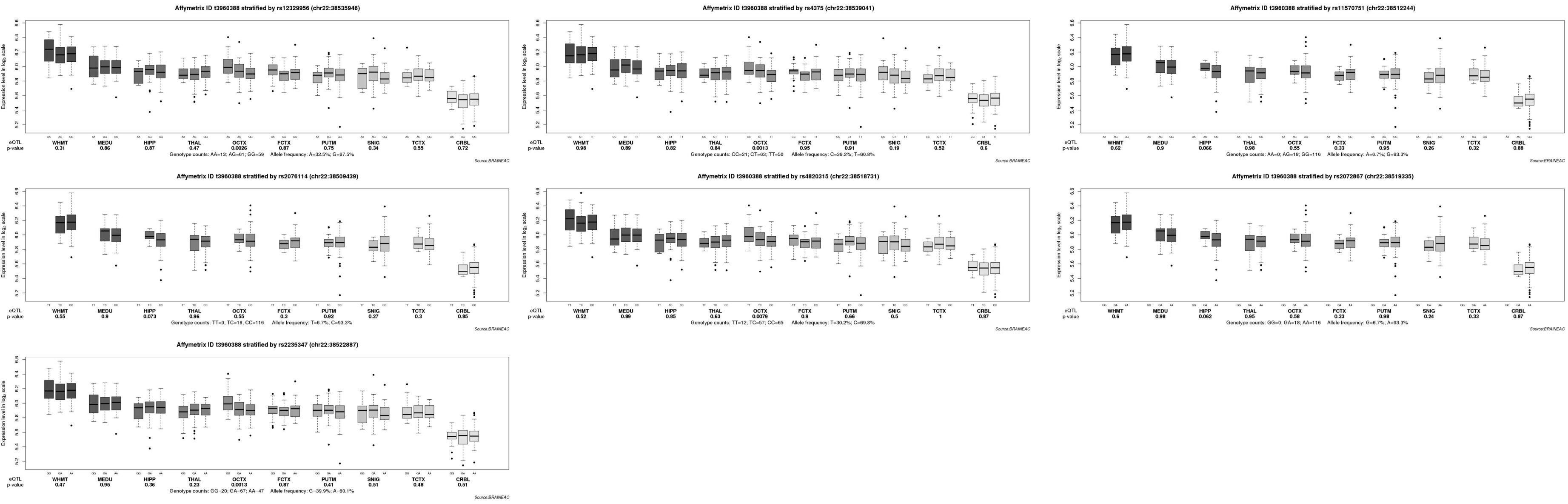

C. FBXO7 expression stratified by different variations

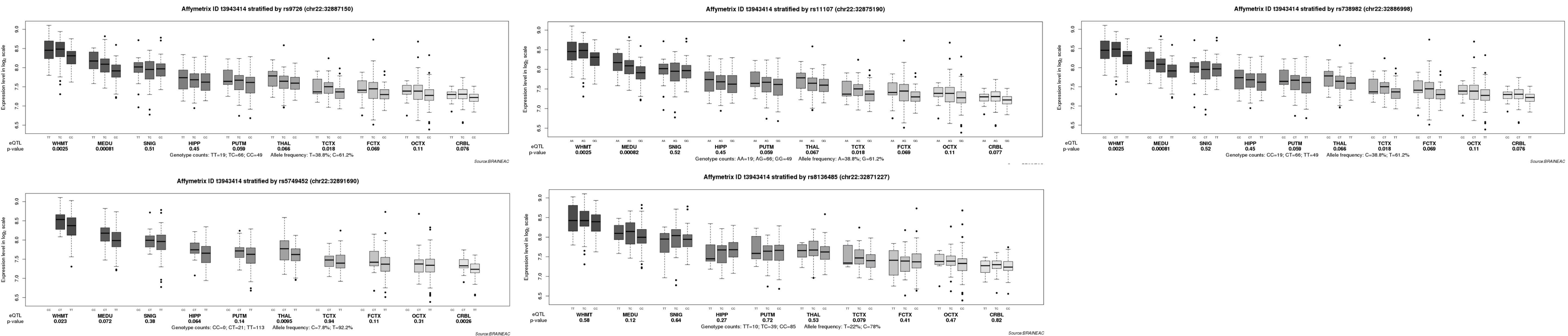

Supplementary Fig.2. The effect of different variants on the expression levels of three transcripts (ATP13A2, PLA2G6 and FBXO7) in Braineac database. Box plot of mRNA expression levels for these three genes in ten brain regions. Abbreviations: THAL, thalamus; FCTX, frontal cortex; SNIG, substantia nigra; TCTX, temporal cortex; MEDU, medulla inferior olivary nucleus; HIPP, hippocampus; OCTX, occipital cortex; PUTM, putamen; WHMT, intralobular white matter; CRBL, cerebellum.
